# Association between metabolic score for visceral fat and psoriasis: findings from NHANES

**DOI:** 10.1186/s40001-025-03002-7

**Published:** 2025-08-12

**Authors:** Xinyi Shao, Yi Ou, Qian Liu, Yidian Fu, Yan Pan, Aijun Chen, Genlong Bai, Jingbo Zhang

**Affiliations:** 1 Department of Dermatology, The First Affiliated Hospital of Chongqing Medical University, Chongqing, China; 2 Graduate School of Hebei Medical University, Shijiazhuang, 050017 Hebei China; 3 Chongqing Medical University, Chongqing, China

**Keywords:** Metabolic score for visceral fat, Psoriasis, Cross-sectional study, NHANES

## Abstract

**Background:**

Psoriasis is a persistent inflammatory skin condition. Several studies have revealed that obesity significantly contributes to both the initiation and advancement of psoriasis. The metabolic score for visceral fat (METS-VF) represents an innovative measure designed to forecast visceral obesity, integrating factors such as insulin resistance metabolic score, waist-to-hip ratio (WHR), age, and gender. The present study aimed to investigate the association between METS-VF and psoriasis prevalence, using information gathered from the National Health and Nutrition Examination Survey (NHANES).

**Methods:**

This study utilized the data from a nationally representative cohort of 8023 adults from NHANES from 2003–2006 to 2009–2014, of which 234 declared a psoriasis history. Multivariate logistic regression analysis and restricted cubic spline (RCS) analyses were used to investigate the association between METS-VF and psoriasis, followed by subgroup analysis to identify populations that may exhibit higher sensitivity.

**Results:**

After adjusting for confounding variables, the results of the multivariate logistic regression analysis showed a significant positive association between METS-VF and the risk of psoriasis. One-unit increasement in METS-VF corresponded to a 47% rise in psoriasis risk (odds ratio [OR] = 1.47, 95% confidence interval [CI] = 1.10–1.96). Finally, the results were uniform across all subgroups (*P*_for interaction _> 0.05). The results from the RCS analysis indicated a notable linear association.

**Conclusion:**

This research indicated that elevated levels of METS-VF are linked to a higher occurrence of psoriasis, suggesting the potential of METS-VF as a predictive anthropometric index for assessing the risk of developing psoriasis.

**Supplementary Information:**

The online version contains supplementary material available at 10.1186/s40001-025-03002-7.

## Introduction

Psoriasis is a skin disease which characterized by red, disfiguring skin plaques accompanied by involvement of multiple organs including joints, cardiovascular system, kidneys, and metabolism [1, 2]. It can lead to a substantial physical and psychological burden to patients. According to a recent epidemiological study, it impacts more than 60 million adults and children globally, resulting in a considerable societal burden [3, 4]. The exact cause of psoriasis remains not fully understood, but it is considered a multifactorial disease, with both genetic and environmental factors contributing to its development. Numerous studies have identified that environmental risk factors such as pharmaceuticals, tobacco use, alcohol consumption, and infections may influence the onset of psoriasis by interacting with both genetic susceptibility and the immune system’s response [5].

Obesity frequently accompanies psoriasis and may exacerbate its development and severity by possibly boosting inflammation. Previous studies have established that obesity can enhance the connection between inflammation and metabolic syndrome (MetS). In addition, obesity markedly elevates the risk of developing cardiovascular disease (CVD). In a similar vein, obesity contributes to both the initiation and advancement of psoriasis, primarily by fostering inflammatory processes [6–9]. Most prior studies have elucidated the connection between psoriasis and obesity by utilizing body mass index (BMI) [10–12]. However, BMI may not provide a detailed assessment of adipose tissue distribution [13, 14]. Other indices like waist circumference (WC), hip circumference (HC), waist–height ratio (WHtR) and WHR also have similar drawbacks with BMI. Previous study suggested that compared with other ectopic fat deposition sites, visceral fat (VF) was more strongly associated with cardiovascular metabolic risk factors [15, 16]. Unlike subcutaneous fat, visceral adipose tissue (VAT) exhibits heightened metabolic activity and is more prone to producing harmful metabolic products such as leptin and proinflammatory cytokines [17]. Therefore, a more reliable approach is needed to accurately reflect the level of visceral adiposity in order to predict psoriasis more effectively.

METS-VF, which is calculated based on the metabolic score for insulin resistance (METS-IR), WHR, age, and gender, is a novel index for predicting visceral obesity. Previous study has found that METS-VF is a novel alternative indicator for estimating VAT, with better performance compared to other alternative VAT indices [18]. Related research indicated that the METS-VF index is effective in identifying VF levels associated with psoriasis risk factors, including diabetes, hypertension, and nonalcoholic fatty liver disease (NAFLD) [18–20]. Currently, the association between the METS-VF index and psoriasis remains ambiguous. To address this knowledge gap, this study intends to perform a cross-sectional analysis utilizing NHANES data to explore the relationship between METS-VF and psoriasis.

## Methods

### Data source and study population

This study utilized data obtained from the NHNAES database, a survey that evaluates the nutritional and health status of citizens across the United States. The research protocol was sanctioned by the Research Ethics Review Board at the National Center for Health Statistics (NCHS), and informed consent was obtained from all participants through the signing of consent forms. The present prospective cohort study involved screening and analyzing data from 5 cycles between 2003–2006 and 2009–2014.

**Fig. 1:**
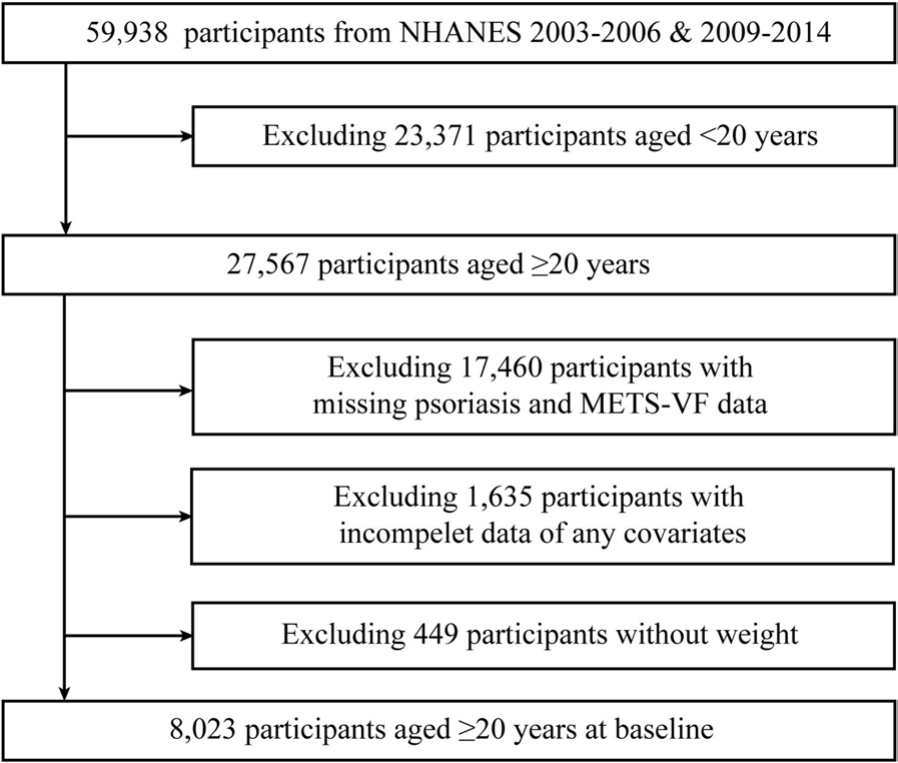
Flowchart of the inclusion and exclusion process for participants in the NHANES database from 2003 to 2006 and 2009 to 2014. *METS-VF* metabolic score for visceral fat, N*HANES* National Health and Nutrition Examination Survey

To maintain the precision and dependability of the findings, particular exclusion criteria were implemented, including (1) individuals < 20 years of age (*n* = 23,371); (2) individuals who did not know if they had psoriasis (*n* = 3,503); (3) missing related measurements for METS-VF (*n* = 13,957); (4) missing measurements of relevant covariates (*n* = 2,084). Finally, the sample size included in the study was 8023 participants after screening **(**Fig. 1**)**.

### Definitions of psoriasis

Certified dermatologists diagnosed psoriasis through a thorough morphological assessment of distinctly outlined red plaques accompanied by silvery scales. Data were gathered using a questionnaire administered by the interviewer. The survey inquired of participants, “Have you ever been told by a doctor or healthcare provider that you have psoriasis?” Individuals who provided an affirmative response were categorized as having psoriasis, whereas those who chose not to respond or indicated they were unsure were classified as not experiencing the condition. The reliability of self-reported psoriasis conditions has been supported by the previous research [21].

### Definition of METS-VF

To calculate METS-VF, multiple metabolic indicators like WHR, METS-IR, gender, and age are integrated. As per the description of the NHANES dataset, laboratory analyses yielded information regarding high-density lipoprotein cholesterol (HDL-C), fasting blood glucose (FBG), and triglycerides (TG). In contrast, measurements such as BMI, height, waist circumference (WC) and were evaluated utilizing mobile screening devices. The computation of METS-VF and other indicators are articulated by the following formula:

$${\text{WHtR}} = \frac{{{\text{WC}}}}{{{\text{Height}}}}$$

$${\text{METS}} - {\text{IR}} = \frac{{\ln (2 \times {\text{FBG}} + {\text{TG}}) \times {\text{BMI}}}}{{\ln ({\text{HDL}} - C)}}$$

$$\begin{gathered} {\text{METS}} - {\text{VF}} = 4466 + 0.01 \times (\ln ({\text{METS}} - {\text{IR}}))^{3} + 3.329 \hfill \\ \times (\ln ({\text{WHtR}}))^{3} + 0.319 \times {\text{gender}} + 0.564 \times \ln ({\text{age}}) \hfill \\ \end{gathered}$$

Within the METS-VF computation, the numerical representation for gender was assigned as 1 for male participants and 0 for female participants. Participants were divided into four groups (Q1, Q2, Q3, and Q4) based on the METS-VF quartiles, with the Q1 group serving as the reference group.

**Fig. 2:**
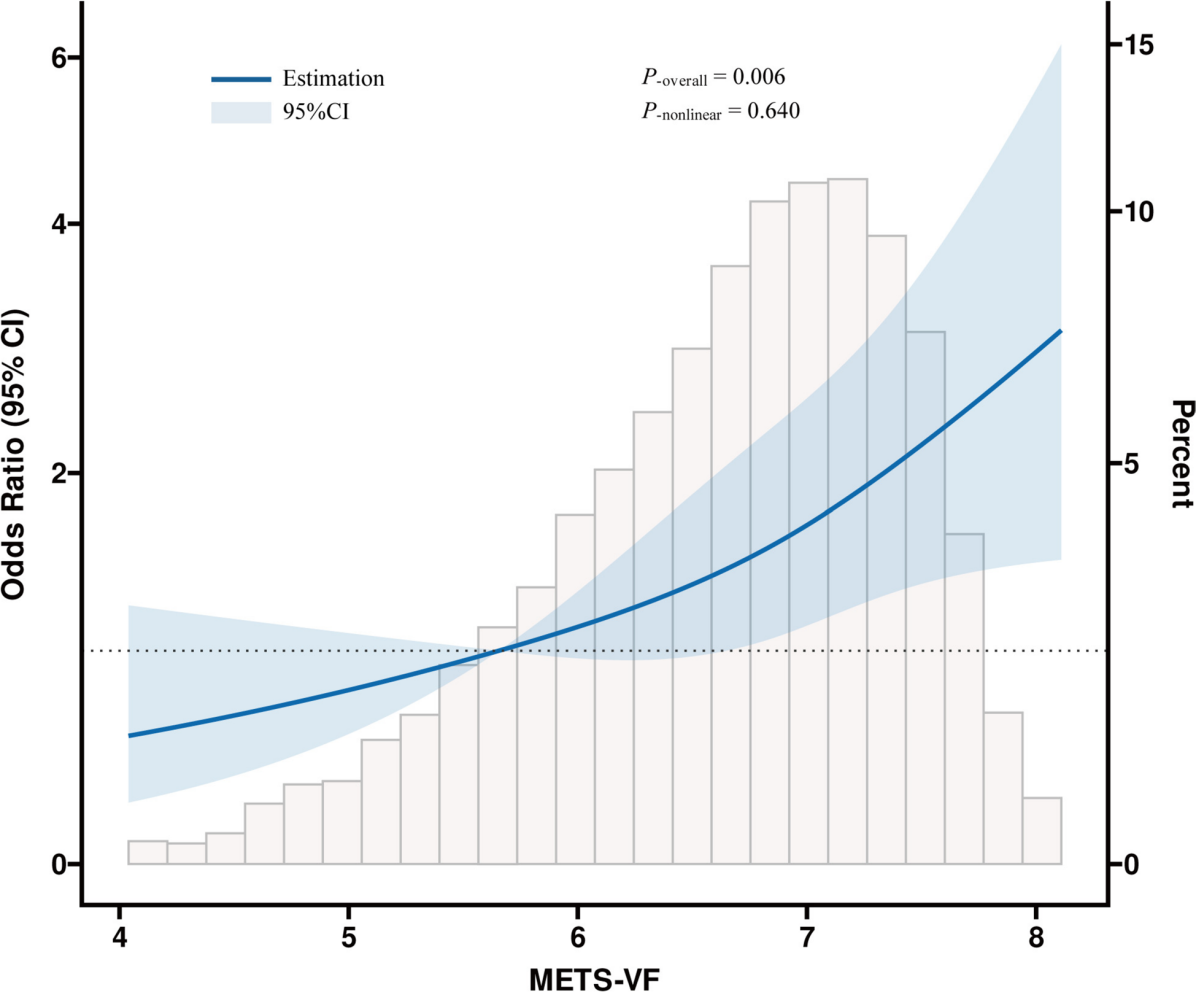
The dose–response associations between the METS-VF and psoriasis. The solid blue line represents the estimates for the association of METS-VF with psoriasis, and shaded area is the 95% CI

### Covariates

In the present study, the following covariates were collected including age, gender, race/ethnicity, education level, poverty income ratio (PIR), marital status, smoking status, alcohol drinking status, history of hypertension, diabetes, CVD, stroke, cancer and survey cycles. Detailed descriptions of the covariate assessments are provided in the Supplementary Materials.

### Statistical analysis

R software (version 4.4.1) and EmpowerStats (version 4.1) software were used to conduct statistical analyses in the present study. Taking into consideration the complex survey design of NHANES, all results were weighted to provide a national estimate of noninstitutionalized US civilian residents. For continuous variables with a normal distribution, means ± standard deviation (SD) was used, and categorical variables were displayed as frequencies (percentages). Continuous variables with normal distributions were analyzed using an independent samples* t* test. For categorical variables, the chi-squared test was used, and Fisher’s exact test was applied if the chi-squared test’s assumptions were not met.

To determine the association between psoriasis risk and METS-VF, a multivariate logistic regression analysis was performed. Model 1 was the basic, unaltered; Model 2 included adjustments for key demographic elements (sex, age, and race). Model 3 incorporated adjustments for all covariates.

In model 3, a regression analysis using RCS was employed to assess the dose–response relationship between psoriasis risk and METS-VF, and threshold effect analysis was conducted to pinpoint critical values. A stratified analysis of the relationship between psoriasis risk and METS-VF was also performed. Statistical significance is indicated by a *P* value less than 0.05 on a 2-tailed test.

## Results

### Baseline characteristics

The study encompassed 8023 participants for final analysis, whose average age was 46.0 ± 16.9 years. Overall, 7802 (97.25%) participants did not have psoriasis while 221 (2.75%) had psoriasis. When compared with nonpsoriasis patients, psoriasis group had a higher METS-VF levels of 6.90 ± 0.66 (*P* < 0.001). Specifically, those suffering from psoriasis exhibited a propensity toward higher age, non-Hispanic white, a higher incidence of hypertension and cardiovascular disease (*P* < 0.05). **(**Table 1**)** Besides, the excluded participants data were listed in the Supplementary materials Table S1.

**Table 1:** Characteristics of Participants in the NHANES datasets 2003–2006 & 2009–2014

Characteristic	Participants^a^			
	Total (*N* = 8023)	Without psoriasis (*N* = 7802)	With psoriasis (*N* = 221)	*P* value
Age, mean ± SD	46.0 ± 16.9	45.93 ± 16.92	49.43 ± 16.10	0.002
LDL, mean ± SD	114.5 ± 35.2	114.44 ± 35.16	115.21 ± 35.5	0.749
METS-VF, mean ± SD	6.7 ± 0.7	6.69 ± 0.74	6.90 ± 0.66	< 0.001
Year cycles, N (%)				0.401
2003–2004	1022 (15.82)	991 (15.70)	31 (19.47)	
2005–2006	1178 (21.71)	1147 (17.10)	31 (20.78)	
2009–2010	2067 (21.54)	2009 (21.56)	58 (22.94)	
2011–2012	1793 (21.94)	1739 (21.96)	54 (21.21)	
2013–2014	1963 (23.49)	1916 (23.68)	47 (17.60)	
Gender, N (%)				0.464
Male	3878 (48.89)	3773 (48.98)	105 (46.12)	
Female	4145 (51.11)	4029 (51.02)	116 (52.05)	
Race and ethnicity^b^, N (%)				0.002
Hispanic	1909 (12.90)	1873 (13.09)	36 (7.18)	
Non-Hispanic white	3798 (69.83)	3662 (69.49)	136 (80.34)	
Non-Hispanic black	1577 (11.09)	1546 (11.21)	31 (7.15)	
Other	739 (6.18)	721 (6.21)	18 (5.33)	
Educational level, N (%)				0.389
Below than high school	1810 (15.69)	1767 (15.75)	43 (13.88)	
High school/equivalent	1774 (22.09)	1724 (20.68)	50 (19.05)	
Greater than high school	4439 (62.23)	4311 (62.07)	128 (67.06)	
Marital status, N (%)				0.806
Married	4251 (56.73)	4135 (56.75)	116 (56.11)	
Never married	1543 (18.82)	1513 (18.82)	30 (16.68)	
Living with partner	709 (8.32)	688 (8.31)	21 (8.66)	
Others^c^	1520 (16.13)	1466 (16.05)	54 (18.56)	
Family PIR, N (%)				0.810
< 1.3	2475 (21.07)	2404 (21.09)	71 (20.16)	
1.3 to < 3.5	2937 (35.87)	2872 (35.92)	65 (34.33)	
≥ 3.5	2611 (43.07)	2526 (42.99)	85 (45.51)	
Smoking status, N (%)				0.051
No	4489 (55.05)	4382 (55.28)	107 (47.73)	
Yes	3534 (44.95)	3420 (44.72)	114 (52.27)	
Alcohol drinking, N (%)				0.558
No	2147 (21.67)	2095 (21.75)	52 (19.45)	
Yes	5876 (78.73)	5707 (78.25)	169 (80.55)	
Hypertension, N (%)				< 0.001
No	5065 (66.15)	4955 (66.61)	110 (51.49)	
Yes	2958 (33.85)	2847 (33.39)	111 (48.51)	
Diabetes mellitus, N (%)				0.603
No	7057 (90.00)	6866 (90.87)	191 (91.92)	
Yes	966 (9.10)	936 (9.13)	30 (8.08)	
CVD ^d^, N (%)				0.002
No	7505 (94.42)	7311 (94.57)	194 (89.70)	
Yes	518 (5.58)	491 (5.43)	27 (10.30)	
Stroke, N (%)				0.551
No	7794 (97.66)	7581 (97.68)	213 (97.12)	
Yes	229 (2.34)	221 (2.32)	8 (2.88)	
Cancer, N (%)				0.313
No	7402 (91.95)	7208 (92.01)	91 (84.70)	
Yes	621 (8.05)	594 (7.99)	27 (9.99)	

*CVD* cardiovascular disease, *LDL* low-density lipoprotein, *METS-VF* metabolic score for visceral fat, *PIR* poverty impact ratio, *SD* standard deviation

^a^Data are presented as an unweighted number (weighted percentage) unless otherwise specified

^b^Race/ethnicity were based on self-reported

^c^ Included participants who was widowed, divorced, or separated

^d^CVD was established based on the presence or absence of coronary heart disease, congestive heart failure, myocardial infarction, or angina

### The association between METS-VF and psoriasis

The multiple regression analyses, including various adjustments to eliminate confounding influences on the correlation, were used to explore the relationship between METS-VF and psoriasis. Both in the unadjusted model 1 and the fully adjusted model 3, it can be inferred that there is a significant positive association **(**Table 2**)**. In fully adjusted covariates model 3, per unit elevation in METS-VF raised the possibility of developing psoriasis by 47% (OR = 1.47, 95% CI = 1.10–1.96). Upon dividing the METS-VF into four quartiles, the highest group demonstrated to be a remarkable 1.08-fold escalation in psoriasis risk morbidity, when compared with the lowest group (OR = 2.08, 95% CI = 1.13–3.82). Furthermore, RCS model was employed to flexibly model and visualize the associations between METS-VF and psoriasis. The findings from the RCS analysis indicate that, following fully adjusted for confounding variables, a notable association can be observed; however, no evidence of a nonlinear relationship was identified. (*P*_overall_ < 0.05, *P*_nonlinear_ > 0.05) (Fig. 2).

**Table 2:** Associations of METS-VF with psoriasis among US adults (*N* = 8023)

	Model 1^a^		Model 2^b^		Model 3^c^	
	OR (95% CI)	*P* value	OR (95% CI)	*P* value	OR (95% CI)	*P* value
Per 1 unit increase	1.43 (2.54, 3.60)	0.0031	1.49 (1.13, 1.97)	0.0059	1.47 (1.10, 1.96)	0.0114
Quartiles						
Q1 (< 6.29)	Ref		Ref		Ref	
Q2 (6.29 to < 6.84)	1.41 (0.83, 2.41)	0.2076	1.45 (0.83, 2.54)	0.1924	1.48 (0.85, 2.56)	0.1700
Q3 (6.84 to < 7.23)	1.17 (0.70, 1.95)	0.5444	1.24 (0.73, 2.10)	0.0101	1.23 (0.71, 2.13)	0.4627
Q4 (≥ 7.23)	2.09 (1.29, 3.38)	0.0036	2.18 (1.26, 3.79)	0.0071	2.08 (1.13, 3.82)	0.0223
*P* for trend	0.0058		0.0101		0.0337	

*CI* confidence interval, *METS-VF* metabolic score for visceral fat, *OR* odds ratio, *Q* quartiles

^a^Model 1: unadjusted model

^b^Model 2: adjusted for age, gender, and race/ethnicity

^c^Model 3: full adjusted model

**Fig. 3:**
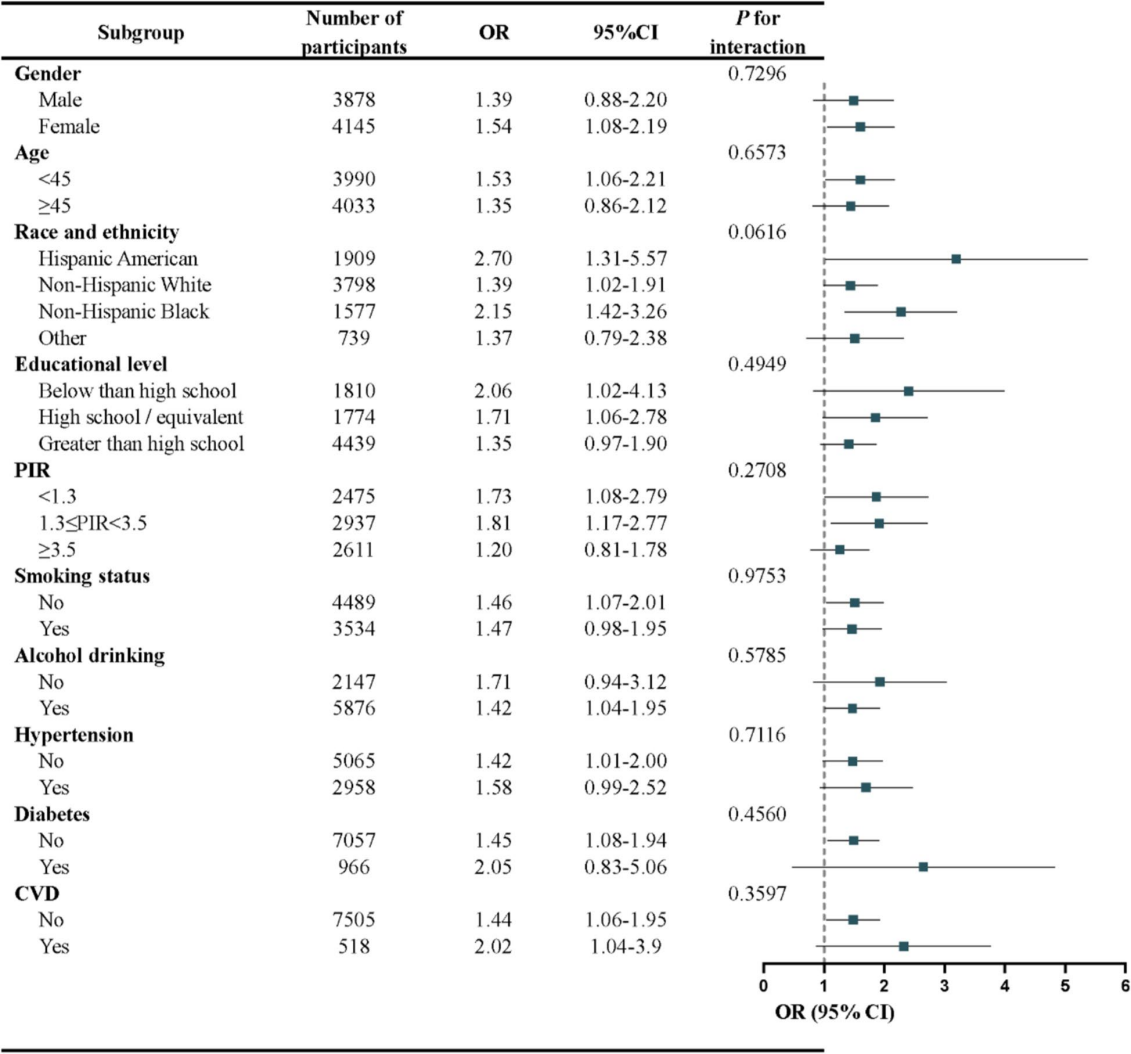
Stratified analysis of the association between METS-VF and psoriasis in adults in the NHANES 2003–2006 and 2009–2014

### Subgroup analysis

To assess the stability of the association between METS-VF and psoriasis within the population, further subgroup analyses were carried out. These analyses were stratified across various factors. The outcomes of these subgroup analyses are presented in Fig. 3. It is important to highlight that, although a statistically significant positive correlation was detected in the majority of subgroups, such significance was not evident in males, older age groups, individuals of other races, those with education levels exceeding high school, families with a higher PIR, smoker, non-alcohol consumers, and those with diabetes. In addition, the findings of this study indicated that the relationship between METS-VF and psoriasis remained consistent across diverse populations (*P* _for interaction_ > 0.05).

## Discussion

Among 8,023 participants in this cross-sectional study, a significant association was identified between the METS-VF index and psoriasis in U.S. adults, showing that individuals with higher METS-VF levels were more likely to develop psoriasis. The correlation was stable across different subgroups, including those based on sex, age, ethnicity, education, alcohol consumption, smoking, cardiovascular disease, hypertension, diabetes, and hyperlipidemia.

However, it could be more pertinent to female, middle- to low-income participants, who drink alcohol, and participants without diabetes. These results may be related to sex hormones, differences in daily health management caused by income. Previous study suggested that most immune cells express estrogen receptors and may respond to estrogen stimulation. Androgen regulate inflammation as well by targeting immune cells to attenuate inflammation[22, 23]. And the association between low to medium PIR and abdominal fat accumulation also has been demonstrated by previous studies, poverty leads to cheap food choices, resulting in overconsumption and obesity among households and consumers[24]. The reasonable mechanism for the relationship between alcohol consumption and psoriasis involves chronic systemic inflammation in individuals who consume alcohol, leading to an increase in inflammatory cytokines associated with psoriasis (interleukin [IL] 1 β, IL-6, IL-8, IL-12, IL-17, and IL-23, as well as interferon-γ, tumor necrosis factor [TNF]-α)[25]. Our study also suggested that the association appears to be more pronounced in participants without diabetes, which indicted multiple variables may lead to disease development simultaneously.

There is a long-standing history of studies examining the connection between psoriasis and VF. Goolam et al.’s observational study identified a notable link between psoriasis and VF, showing a 1.56 ratio of abnormal VF in those with psoriasis [26]. A similar conclusion was reached by Ataseven A et al. [27] Another study observed that individuals with psoriasis exhibit an unfavorable body composition, characterized by increased visceral and ectopic liver fat and reduced thigh muscle volume, which is linked to a higher risk of cardiometabolic diseases [28].

METS-VF is a new alternative indicator that merges basic anthropometric measurements with experimental indicators, in contrast to direct imaging techniques like computed tomography and magnetic resonance imaging for assessing VF. Previous research found that METS-VF was more effective than traditional VF measures, such as WC, BMI, WHR, and visceral adiposity index [18, 29]. There are several studies that has shown that METS-VF can serve as a reliable indicator for CVD, diabetes, hypertension, NAFLD, and chronic kidney disease (CKD) [30–34]. Our study uniquely examines the relationship between METS-VF index and psoriasis, as compared to previous research. Our study also evaluated the effectiveness of METS-VF in detecting the prevalence of psoriasis in the current population compared with traditional obesity index and other visceral obesity surrogate markers.

The exact biological mechanisms connecting VF and psoriasis need to be clarified for full understanding, but several potential explanations are available. Numerous investigations have highlighted that the reciprocal connection between VF and psoriasis arises from a shared pro-inflammatory condition [35–37]. Adipose tissue has been identified not only as an active endocrine organ, but also an immune organ that produces varied immune cells and inflammatory-related cytokines such as TNF-α, leptin, and IL-6, which exacerbate the inflammatory processes in psoriasis. In addition, excess adipose tissue contributes to gut dysbiosis, which affects the production of short-chain fatty acids and increases intestinal permeability [38]. This allows pro-inflammatory substances to enter the bloodstream, further exacerbating systemic inflammation and triggering psoriasis flare-ups [39]. Furthermore, superabundant VF-induced changes in gene expression affect critical immune cells, such as Treg lymphocytes and macrophages [6]. These changes contribute to immune dysregulation, which enhances the inflammatory response in both the skin and other systems.

﻿Our research exhibits numerous strengths. It pioneers the evaluation of the association between METS-VF and the prevalence of psoriasis among American adults. The substantial sample size enhances the precision and reliability of our estimates. Our findings reveal a persistent positive correlation between METS-VF and psoriasis incidence, confirming this association is not coincidental. Moreover, we accounted for confounding factors based on demographic features and chronic illness conditions. The present study also performed subgroup analyses to further explore the connection between METS-VF and psoriasis across diverse populations, highlighting the necessity for more targeted psoriasis prevention strategies.

Nonetheless, this study is not devoid of limitations. The cross-sectional design employed does not permit the establishment of a causal relationship between METS-VF and psoriasis due to the absence of a temporal dimension. To substantiate these findings and explore the potential role of METS-VF in psoriasis management and prevention, additional long-term studies are essential. In addition, the NHANES contains psoriasis data ascertained by self-reported data. Self-reported questionnaires may lead to recall bias in psoriasis diagnosis. In order to more accurately estimate the prevalence of psoriasis, future research may need to consider conducting face-to-face dermatological examinations or whole-body photography to detect psoriasis in other undiagnosed participants. And the widely recognized formula used in this study to evaluate visceral fat still cannot reflect the detailed distribution of visceral fat. METS-VF is an indirect indicator for evaluating visceral fat, providing a practical evaluation method for daily life and clinical practice. Furthermore, despite controlling multiple covariates, unmeasured factors such as dietary patterns, physical activity levels, and environmental influences might influence the outcomes. And the study primarily involved the general the United States population, necessitating caution when applying the findings to other ethnic groups. Lastly, as an emerging index of body composition, the clinical application and broad adoption of METS-VF necessitate further investigative backing.

## Conclusion

This research has established a notably positive association between METS-VF and the incidence of psoriasis. Notably, an increasing level of METS-VF is accompanied by a proportional rise in the incidence of psoriasis. Future studies should pay more attention to adipose tissue distribution, particularly VF, to assess its impact on the risk of psoriasis, and to provide personalized health guidance.

## Supplementary Information

Additional file 1

## Data Availability

The data employed in this research is available at this website: https://www.cdc.gov/nchs/nhanes/.

## Abbreviations

AUC: Area under the ROC curve
BMI: Body mass index
CI: Confidence interval
CKD: Chronic kidney disease
CVD: Cardiovascular disease
FBG: Fasting blood glucose
HC: Hip circumference
HDL-C: High-density lipoprotein cholesterol
IL: Interleukin
LAP: Lipid accumulation product
METS-IR: The metabolic score for insulin resistance
METS-VF: Metabolic score for visceral fat
MetS: Metabolic syndrome
NAFLD: Non-alcoholic fatty liver disease
NCHS: The National Center for Health Statistics
NHANES: National Health and Nutrition Examination Survey
OR: Odds ratio
PIR: Poverty income ratio
RCS: Restricted cubic spline
SD: Standard deviation
TG: Triglycerides
TNF: Tumor necrosis factor
VAI: Visceral adiposity index
VAT: Visceral adipose tissue
VF: Visceral fat
WC: Waist circumference
WHR: Waist-to-hip ratio
WHtR: Waist-height ratio
