# Supplementary material for: Association between metabolic score for visceral fat and psoriasis: findings from NHANES
Source: Eur J Med Res. 2025 Aug 12;30:739. doi: 10.1186/s40001-025-03002-7 (PMC12341227; doi:10.1186/s40001-025-03002-7)
Supplement: Supplementary file 1 — Additional file 1 [file 40001_2025_3002_MOESM1_ESM.docx]

**Supplementary materials**

**Detailed definition of covariates**

Participants self-reported their race/ethnicity based on predefined categories, including Mexican American, non-Hispanic Black, non-Hispanic White, and other races and ethnicities (American Indian or Alaska Native, Native Hawaiian or Pacific Islander, and non-Hispanic Asian). Education level is classified into three categories: high school or lower, some college, and college graduate or higher. Household income is stratified into three tiers based on the PIR: low income (≤1.3), middle income (>1.3 to 3.5), and high income (>3.5). Marital status is categorized into four classifications: married, unmarried, living with a partner, and other (which encompasses individuals who are widowed, divorced, or separated). The determination of alcohol drinking status was based on following survey inquiry: “In any 1 year, have you had at least 12 drinks of any type of alcoholic beverage?” Participants who responded affirmatively were classified as alcohol drinkers. Smoking status was assessed through the question: “Have you smoked at least 100 cigarettes in your entire life?” Those who answered “yes” were identified as smokers. Hypertension was defined as self-reported diagnoses hypertension or using antihypertensive medication or average systolic blood pressure > 140 mmHg and/or average diastolic blood pressure > 90 mmHg. Diabetes mellitus was classified based on self-reported diagnosis, HbA1c levels ≥ 6.5%, or the use of insulin. Cardiovascular disease (CVD) was established based on the presence or absence of coronary heart disease, congestive heart failure, myocardial infarction, or angina.

Table S1: Baseline characteristics of included and excluded population.

| **Characteristic** | **Participants ^a^** |  |  | |
| --- | --- | --- | --- | --- |
|  | **Include**  (N=8023) | **Exclude**  (N=19544) | | ***P* value** |
| **Age, mean ±SD** | 46.0±16.9 | 50.7±18.8 | | <0.001 |
| **Gender, N (%)** |  |  | | 0.90 |
| Male | 3878 (48.3) | 9431 (48.3) | |  |
| Female | 4145 (51.7) | 10113 (51.7) | |  |
| **Race and ethnicity ^b^, N (%)** |  |  | | <0.001 |
| Mexican American | 1909 (23.8) | 4550 (23.3) | |  |
| Non-Hispanic Black | 3798 (47.3) | 8875 (45.4) | |  |
| Non-Hispanic White | 1577 (19.7) | 4294 (22.0) | |  |
| Other | 739 (9.2) | 1825 (9.3) | |  |
| **Educational level, N (%)** |  |  | | <0.001 |
| High school or less | 1810 (22.6) | 5425 (27.8) | |  |
| Some college | 1774 (22.1) | 4574 (23.5) | |  |
| College graduate or higher | 4439 (55.3) | 9495 (48.7) | |  |
| **Marital status ^c^, N (%)** |  |  | | <0.001 |
| Married | 4251 (53.0) | 9960 (51.0) | |  |
| Never married | 1543 (19.2) | 3497 (17.9) | |  |
| Living with partner | 709 (8.8) | 1342 (6.9) | |  |
| Others ^c^ | 1520 (18.9) | 4721 (24.2) | |  |
| **Family PIR, N (%)** |  |  | | <0.001 |
| <1.3 | 2475 (30.8) | 5673 (32.6) | |  |
| 1.3 to<3.5 | 2937 (36.6) | 6469 (37.2) | |  |
| ≥3.5 | 2611 (32.5) | 5258 (30.2) | |  |
| **Smoking status, N (%)** |  |  | | <0.001 |
| No | 4489 (56.0) | 10447 (53.5) | |  |
| Yes | 3534 (44.0) | 9076 (46.5) | |  |
| **Alcohol drinking, N (%)** |  |  | | <0.001 |
| No | 2147 (26.8) | 4690 (29.8) | |  |
| Yes | 5876 (73.2) | 11059 (70.2) | |  |
| **Hypertension, N (%)** |  |  | | <0.001 |
| No | 5065 (63.1) | 11110 (56.9) | |  |
| Yes | 2958 (36.9) | 8420 (43.1) | |  |
| **Diabetes, N (%)** |  |  | | <0.001 |
| No | 7057 (88.0) | 16579 (84.8) | |  |
| Yes | 966 (12.0) | 2965 (15.2) | |  |
| **CVD ^d^, N (%)** |  |  | | <0.001 |
| No | 7505 (93.5) | 17627 (90.2) | |  |
| Yes | 518 (6.5) | 1905 (9.8) | |  |
| **Stroke, N (%)** |  |  | | <0.001 |
| No | 7794 (97.1) | 18675 (95.7) | |  |
| Yes | 229 (2.9) | 834 (4.3) | |  |
| **Cancer, N (%)** |  |  | | <0.001 |
| No | 7402 (92.3) | 17589 (90.1) | |  |
| Yes | 621 (7.7) | 1926 (9.9) | |  |

Abbreviations: CVD: cardiovascular disease; PIR: poverty impact ratio; SD: standard deviation.

^a^ Data are presented as unweighted number (weighted percentage) unless otherwise specified.

^b^ Race and ethnicity were self-reported.

^c^ Included widowed, divorced, or separated.

^d^ CVD was established based on the presence or absence of coronary heart disease, congestive heart failure, myocardial infarction, or angina.
